# Prey of killer whales (*Orcinus orca*) in Iceland

**DOI:** 10.1371/journal.pone.0207287

**Published:** 2018-12-12

**Authors:** Filipa I. P. Samarra, Manuela Bassoi, Julie Béesau, Margrét Ó. Elíasdóttir, Karl Gunnarsson, Marie-Thérèse Mrusczok, Marianne Rasmussen, Jonathan N. Rempel, Baldur Thorvaldsson, Gísli A. Víkingsson

**Affiliations:** 1 Marine and Freshwater Research Institute, Skúlagata, Reykjavík, Iceland; 2 Sea Mammal Research Unit, Scottish Oceans Institute, University of St Andrews, Fife, St Andrews, United Kingdom; 3 Elding Whale Watching, Ægisgardur, Reykjavik's Old Harbour, Reykjavík; 4 Whales Hauganes ehf., Hafnargata, Hauganesi, Iceland; 5 Orca Guardians Iceland, Hrannarstigur, Grundarfjörður, Iceland; 6 Láki Tours, Nesvegur, Grundarfjörður, Iceland; 7 Húsavík Research Centre, University of Iceland, Hafnarstétt, Húsavík, Iceland; 8 Special Tours, Ægisgarður, Reykjavík’s Old Harbour, Reykjavík, Iceland; 9 Iceland Pro Cruises, Ármúli, Reykjavík, Iceland; Hawaii Pacific University, UNITED STATES

## Abstract

Killer whales have a cosmopolitan distribution and as a species are generalists, feeding on a variety of prey. However, local populations tend to specialise on specific prey types. In Icelandic waters, killer whales are generally associated with herring and, thus, have been presumed to be herring specialists. However, recent studies suggest a more complex foraging ecology, possibly including a mosaic of strategies. With increased observational effort in recent years due to research and whale-watching activities, there have been several reports of interactions with different prey, including confirmed predation events. In this study we aimed to summarise the range of potential prey of killer whales observed in Icelandic waters. We report on 12 previously unpublished accounts and review 15 accounts published in the scientific literature or local newspapers, making a total of 27 events where killer whales were observed interacting with actual or potential prey. Thirteen different species, including birds (n = 1), cephalopods (n = 1), fish (n = 5) and marine mammals (n = 6), are reported, although herring is by far the species that killer whales are most often observed interacting with. This study provides the first summary of actual and suspected killer whale prey in Icelandic waters, and contributes towards our understanding of this population’s prey preferences. However, describing the diet of individuals/groups was not possible and this study points to a need for continued monitoring to understand the intricacies of killer whale foraging behaviour in this area.

## Introduction

Killer whales (*Orcinus orca*, Linnaeus, 1758), as a species, have been reported to predate on numerous prey including fish, marine mammals, cephalopods, marine turtles and birds [1, 2]. However, several populations specialize on particular prey types and ecotypes diverging in diet, morphology, genetics and behaviour have been recognised in the North Pacific and Antarctica (e.g. [3–9]). Nevertheless, killer whales with an apparent mixed diet that includes fish and marine mammals have been reported in several locations [10, 11]. Foraging ecology is tightly related to the social organization, life history and behaviour of killer whales [12] and, ultimately, may play a major role in the social segregation and genetic divergence of populations [13, 14]. Understanding the foraging ecology of each population is relevant to assess both their conservation status and role in marine ecosystems.

In the Northeast Atlantic, two killer whale ecotypes have been suggested based on analyses of morphological traits, stable isotope ratios and tooth wear from museum and stranded specimen; a generalist Type 1, that presumably feeds upon fish and to some extent seals, and a marine mammal specialist Type 2 [15]. High variance in nitrogen stable isotope ratios within Type 1 suggested individual and/or group variation in the proportion of prey types consumed [15]. A subsequent genetic study identified three significantly differentiated populations overlapping spatially with the distribution of Atlantic herring (*Clupea harengus*, Linnaeus, 1758), Atlantic mackerel (*Scomber scombrus*, Linnaeus, 1758) and Atlantic bluefin tuna (*Thunnus thynnus*, Linnaeus, 1758), on which some members of each population had been seen predating [16]. Population A included herring-feeding individuals that were sampled within the ranges of the Norwegian, Icelandic and North Sea herring stocks. Population B included individuals sampled from the North Sea to the West coast of Iceland that appeared to spatially associate with the distribution of the Northeast Atlantic mackerel stock, including one individual sampled from a trawler fishing boat whilst feeding on mackerel discards. Finally, population C was composed solely of individuals sampled in the Canary Islands and in the Strait of Gibraltar, the latter known to target tuna (e.g., [17]).

Killer whales have long been known to occur around Iceland, particularly on seasonal herring grounds, where killer whales are observed feeding upon herring [18]. Although initially suggested to be a population of herring-specialists [18], genetic studies suggested that killer whales sampled in Icelandic waters belong to two separate populations: population A, which associates with herring and population B, which is thought to associate with mackerel [16]. Furthermore, recent studies showed that killer whales occurring in Icelandic coastal waters have different movement and foraging strategies. While some individuals appear to specialise on herring year-round, others prey on it only seasonally or even opportunistically and include other unknown, higher trophic level prey in their diet [19]. Indeed, a few individuals occur regularly in herring overwintering grounds in Iceland but in spring and summer travel to Scotland where they have been seen feeding upon seals [19–21]. Early reports of feeding upon marine mammals and seabirds [22] indicated that prey targeted by killer whales in Iceland extended beyond herring. Nevertheless, target prey species remained relatively unknown. Thus, there is still little understanding of the prey preferences of Icelandic killer whales as well as the potential existence of different ecotypes in these waters.

In recent years, there has been an increase in whale-watching in Iceland, which now takes place in several locations across the country [23]. This has led to increased numbers of killer whale encounters outside herring grounds and interactions with other potential prey have been reported more frequently. In this study, we report on recent unpublished observations of killer whale interactions with potential prey and review available information from published sources to provide a summary of potential prey targeted by killer whales in Icelandic waters. This study aimed to increase knowledge of the prey of Icelandic killer whales, to contribute towards an assessment of the species’ role within this ecosystem.

## Materials and methods

All observations from whale-watching vessels were conducted in accordance with local whale watching guidelines. All field research was carried out in compliance with local regulations and under an institutional permit for the Marine and Freshwater Research Institute, Reykjavík.

Sightings of killer whales interacting with various species in Icelandic waters were compiled from several sources, including previously unpublished reports from dedicated research and commercial whale-watching operations, as well as published accounts on newspapers or other scientific and non-scientific published literature. All observations were summarised in Table 1. Previously published reports are briefly mentioned in the results, along with detailed accounts of recent, unpublished observations. The reported times of events are all in local time (UTC). Observations were conducted opportunistically onboard whale-watching boats or from dedicated research vessels. When available, photographs were used to identify the individual killer whales involved in each incident. Individuals were compared with an existing photo-identification catalogue [24], to understand if those individuals had been observed consuming other prey before.

**Table 1:** Summary of interactions between killer whale and other species in Icelandic waters. Prey is divided into four types: birds, cephalopods, fish and marine mammals. Date is given as dd/mm/yyyy when available or by the month and year. When available, information on location of events is provided as approximate locations or exact coordinates. Approximate and exact locations are plotted in Fig 1, unless the location information provided was too inaccurate, such as “East of Iceland” (see events #15 and 16).

Prey type	Event #	Date	Prey species	Description	Location	Source
Birds	1	February 1986	Common eider (*Somateria mollissima*)	One kill of a male eider	Berufjörður (E Iceland)	this study
	2	18/04/1998	“	Killed at least two birds	Reykjavík (SW Iceland)	this study
	3	12/09/2003	“	Killed at least 30 birds, after severely injuring a seal that appeared to escape	Ísafjarðardjúp (NW Iceland)	[25]
	4	28/11/2006	“	Three killer whales (1 adult male, 1 female and a calf) swimming along the coast, near the rocks and repeatedly surfacing in the middle of eider groups	Djúpivogur (E Iceland)	[26]
	5	29/11/2010	“	Four killer whales swimming close to the rocky shore repeatedly attacking eider groups; one whale followed a bird all the way to the harbour pier where it caught it	Berufjörður (E Iceland)	[27]
	6	16/11/2014	“	Apparently killed two eiders	Grundarfjörður (W Iceland); 64.98° N; 23.37° W	this study
Cephalopods	7	1967	Squid (unknown spp.)	Stomach contents of a killer whale caught by Norwegian whalers	Offshore waters (E Iceland)*	[28]
Fish	8	1932-present	Herring (*Clupea harengus*)	-	Various locations around Iceland and in offshore waters	(e.g., [15, 16, 19, 20, 21, 22, 18, 28, 29])
	9	03/03/2014	Lumpfish (*Cyclopterus lumpus*)	Killed one fish	Kolgrafafjörður (W Iceland); 64.99° N; 23.09° W	this study
	10	11/06/2016	“	One male lumpfish tossed in the air	Off Snæfelsnes (W Iceland); 64.90° N; 24.05° W	this study
	11	15/07/2015	Salmon (*Salmo salar*)	One fish tossed in the air	Vestmannaeyjar (S Iceland); 63.40° N; 20.48° W	this study
	12	27/04/1978	Atlantic halibut (*Hippoglossus hippoglossus*)	Depredation from longline fishing, leading to fishermen having to abandon fishing	-	[30]
	13	May 1979	“	Several hundreds of killer whales in the fishing grounds, often taking every Atlantic halibut off the hooks; selectively choosing halibut over redfish (*Sebastes* spp.) or tusk (*Brosme brosme*, Ascanius, 1772)	70–120 nm SW off Garðskaga (SW Iceland)	[31]
	14	13/05/1992	“	Depredation from longline fishing, killer whales taking the fish whole leaving just the heads on the hooks, leading to the fishermen abandoning fishing	-	[32]
	15	04/08/1970	Greenland halibut (*Reinhardtius hippoglossoides*)	Depredation from longline fishing, often the whales took all the Greenland halibut off the hooks	East of Iceland	[33]
	16	25/07/1971	“	Fisheries scientist reporting poor fishery due to killer whale depredation	“	[34]
Marine mammals	17	20/07/1987	Pilot whale (*Globicephala melas*)	A group of pilot whales, observed from a survey aircraft, being chased by killer whales and blood seen in the water	63.22°N, 19.17°W	[35]
	18	03/11/1915	Common minke whale (*Balaenoptera acutorostrata*)	Witnesses report on a recent attack by killer whales on a minke whale; a floating carcass was found two days later and all blubber had been stripped off	Hvalfjörður (SW Iceland)	[36]
	19	1997	“	Minke whaler reports an observation that took place in the early half of the 20^th^ century, of a medium sized minke whale being attacked by killer whales; one of the killer whales is killed by the whalers and stomach contents revealed approximately 70 kg of whale meat, a ventral groove blubber piece >1 m long and 1 ft. wide and the hind part of a harbour porpoise; the whaler remembers also two instances where he found minke whale carcasses in the same location that had clearly been killed by killer whales	Ísafjarðardjúp (NW Iceland)	[37]
	20	22/07/2008	“	Killed but consumption not confirmed	Skjálfandi Bay (NE Iceland)	this study
	21	06/06/1982	Harbour porpoise (*Phocoena phocoena*)	Minke whaler reports once seeing two killer whales taking a harbour porpoise between them and tearing it apart	-	[38]
	22	27/06/2012	“	Killed but consumption not confirmed	Skjálfandi Bay (NE Iceland); 66.05°N; 17.67° W	this study
	23	23/09/2017	“	Killed and presumably consumed	Eyjafjörður (N Iceland); 65.97°N; 18.31° W	this study
	24	17/12/2017	White-beaked dolphin (*Lagenorhynchus albirostris*)	Killed and presumably consumed	Faxaflói Bay (SW Iceland); 64.2° N; 22.03° W	this study
	25	21/02/1967	Grey seal (*Halychoerus grypus*)	Two stranded killer whales with grey seals in their stomach, one with eaten parts of five seals and the other with at least two; the seals had been bitten across and, in some cases, only the head was taken off	Breidafjörður (W Iceland)	[39]
	26	07/07/1993	“	Attacked and presumably consumed	Breidafjörður (W Iceland)	this study
	27	27/06/2012	Harbour seal (*Phoca vitulina*)	Killed but consumption was not confirmed	Skjálfandi Bay (NE Iceland)	this study

**Fig 1:**
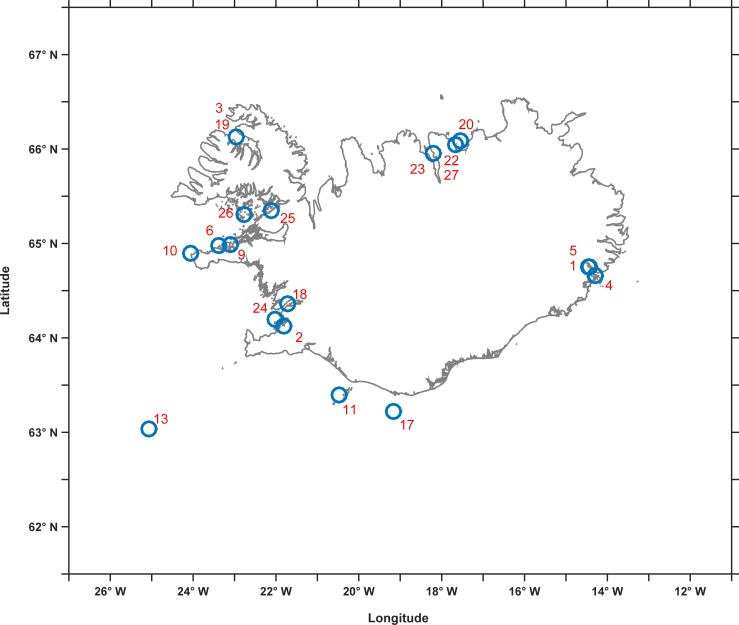
Map of approximate locations of events of killer whale interactions with the different prey species described in this study. Numbers correspond to events listed in Table 1. If more than one event occurred in the same location the numbers are listed vertically.

* This individual was caught in a region considered by the authors as waters off Iceland (East of Iceland and adjacent waters of the Norwegian Sea), and it lies about half way between Iceland and Norway. It is not plotted in Fig 1 as this figure shows only locations closer to the coast.

Published records of interactions between killer whales and other species were obtained from local printed media accessed through a public database (timarit.is). The keyword ‘háhyrningur’ (Icelandic common name for killer whale) and all its possible inflections were used to search the database. The resulting news items included material published between 1880 and 2016 and were investigated to find relevant accounts of killer whales confirmed interacting with other species. Relevant interactions are listed in Table 1 with references to the newspaper and date of publication. Accounts published in the scientific literature were also summarised. Only interactions where killer whales were observed chasing, harassing and attacking or presumably attacking other species, defined as predatory interactions [2], were included. Additional observations of stomach contents or prey handling where the capture was not recorded were also included.

## Results

A total of 13 different potential prey species were identified in this study, including birds, cephalopods, fish and marine mammals (Table 1). Predation events occurred all around Iceland (Fig 1). Twelve recently observed accounts of killer whale interactions with different prey are reported here for the first time (Table 1). The remaining 15 accounts reviewed consisted of 3 events reported previously in the scientific literature and 12 events reported in newspaper articles. Predation and lethal harassment can be difficult to distinguish, particularly from popular accounts, so we included a description in Table 1 of what was in the published literature, without assuming consumption unless clearly observed. For all the previously unpublished observations reported here, we state when consumption of prey was not confirmed. A detailed description of previously unpublished accounts, along with a mention of accounts previously reported in the literature, is given below, separated into four main species groups: 1) birds; 2) cephalopods; 3) fish; and 4) marine mammals.

### Birds

#### Common eider (*Somateria mollissima*, Linnaeus, 1758)

In February 1986, a group of approximately 12 to 15 killer whales, including two adult males, were observed in the inner part of Berufjörður (E Iceland), in Fossárvík (Table 1, event #1). Two relatively small animals were observed surfacing where a flock of common eiders was densely grouped close to the shore. One of the whales grabbed a male eider and took it underwater and then the rest of the eider flock fled into a cove. The bird was not seen again so it was presumed to be consumed. The killer whales disappeared underwater and then came up under the birds only a few meters away from the observers. The eiders all rushed away just before the whales surfaced. Shortly after, the killer whales left the area to join the rest of the group out on the fjord. No photo-identification data were available for this event.

On 18 April 1998, a small group of killer whales was observed hunting, attacking and eating common eiders along the coast of Reykjavík (Table 1, #2). The killer whale group was composed of two adult males, a female with a calf and one subadult male/adult female. The killer whales moved systematically along the coast (approximately 10–100 m from the shore) and surfaced repeatedly in groups of eiders. At least two eiders were observed swallowed and probably more were eaten. Photographs of only the right sides of the whales involved in this event were available but it was not possible to identify any individuals.

Three further accounts of predation on common eiders were reported in newspapers and occurred in Ísafjarðardjúp (NW Iceland) in 2003 (Table 1, #3), Djúpivogur (E Iceland) in 2006 (Table 1, #4) and Berufjörður (E Iceland) in 2010 (Table 1, #5).

On 16 November 2014 at approximately 14:33, two killer whales (identified as IS015 and IS229, [24]) were observed attacking common eiders just outside Grundarfjörður (Table 1, #6). Both individuals were observed lunging out of the water in the locations where eiders were sitting at the sea surface. On two occasions, the bird targeted seemed to have been pulled under the surface. Prey consumption was not confirmed.

### Cephalopods

Predation on cephalopods was reported once in the scientific literature, as part of a list of items found in the stomach contents of killer whales caught by whalers (Table 1, #7).

### Fish

#### Herring (*Clupea harengus*, Linnaeus, 1758)

There are numerous observations of killer whales occurring in seasonal herring grounds, making this by far the prey that killer whales are most commonly observed consuming in Iceland. The first published scientific book mentioning herring as a main prey of killer whales dates back to 1932 and since then there have been numerous studies reporting herring consumption (Table 1, #8). Indeed, killer whales in Iceland were proposed to be herring-specialists [18], which has been supported by recent studies [19, 21].

#### Lumpfish (*Cyclopterus lumpus*, Linnaeus, 1758)

**Fig 2:**
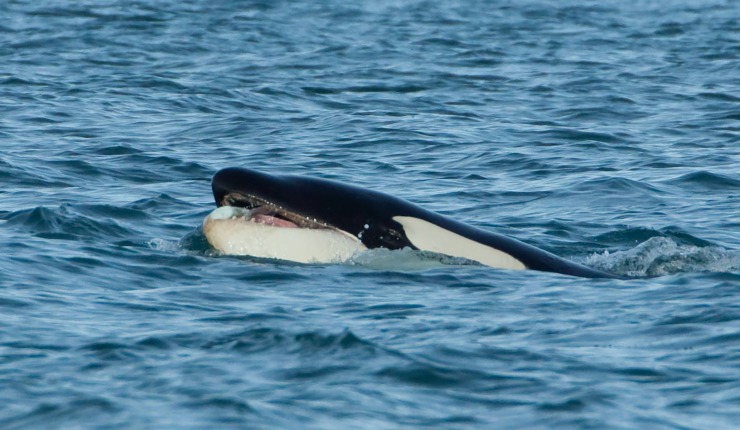
Killer whale with a lumpfish in its mouth. Observed in Kolgrafafjörður (W Iceland) on 3 March 2014 (photograph by W. Jan Strietman).

Three killer whales (identified as IS015, IS113 and IS229, [24]) were observed in the morning of the 3 March 2014, at approximately 11:13, just outside Kolgrafafjörður (Table 1, #9). The whales were tracked from a whale-watching boat following the coastline, heading east. Approximately 1h later, at the entrance of the fjord, individual IS229 was observed with a fish in its mouth (Fig 2). Photographs were later used for identification of the fish species, consulting experts that confirmed it to be a lumpfish. Great Black-backed gulls (*Larus marinus*) and Glaucous gulls (*Larus hyperboreus*) followed the killer whale and attempted to scavenge on pieces of fish from the surface, but consumption of the fish by the whale was not confirmed. A few minutes later, the whale-watching boat departed the area. A dedicated research vessel found the same individuals in that area in the afternoon. At approximately 13:44, one whale was again seen with the head out of the water and holding something in its mouth and once again it was followed by gulls that picked up a piece of what appeared to be fish. However, it was not possible to determine the species on this occasion. The killer whales were then observed travelling very close to a seal haul-out location, closely following the shoreline. However, no interactions were observed even though at least one seal was present in the water.

On 11 June 2016, one animal was observed at approximately 15:36 just off the tip of the Snæfellsnes peninsula (Table 1, #10). The whale was observed lunging often and spyhopped once, followed by a backflip while tossing a lumpfish up with its rostrum. The individual was then seen rolling on its side, no further surface activity was observed and the individual was just milling. Prey consumption was not confirmed. Another five individuals were present in the area but were not interacting with fish. Photographs from this event were available but it was not possible to match the individual involved.

#### Salmon (*Salmo salar*, Linnaeus, 1758)

**Fig 3:**
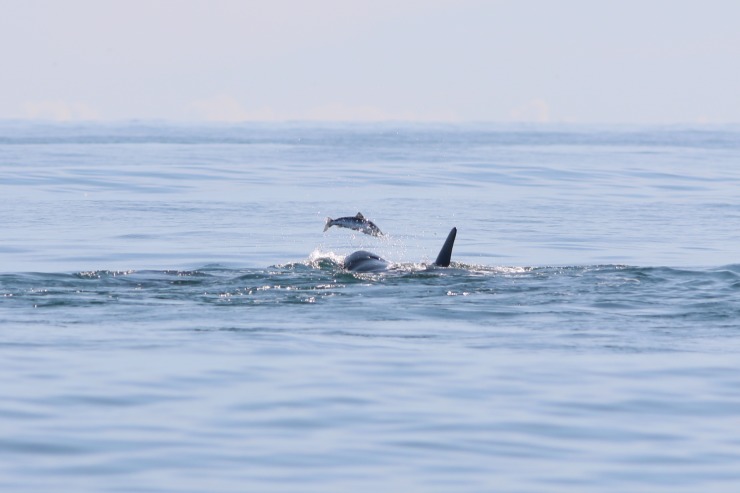
Killer whale tossing a salmon in the air. Observed in Vestmannaeyjar (S Iceland) on 15 July 2015 (photograph by F. Samarra).

On 15 July 2015, at approximately 16:12, a dedicated research vessel was following a group of four killer whales, including two adult females, a calf and one juvenile in Vestmannaeyjar (Table 1, #11). The group was observed swimming erratically at the surface turning sharply as if chasing something underwater. This went on for a few minutes until the whales were seen tossing a fish in the air (Fig 3). Photographs of the event were being collected throughout for photo-identification. Although the fish was observed being tossed a few times, consumption could not be confirmed due to the distance to the whales. Later inspection of the photographs with consultation of experts revealed the fish species to be salmon (*Salmo salar*).

#### Atlantic halibut (*Hippoglossus hippoglossus*, Linnaeus, 1758) and Greenland halibut (*Reinhardtius hippoglossoides*, Walbaum, 1972)

Accounts from newspapers reported depredation from longline fisheries for Atlantic halibut in 1978 (Table 1, #12), 1979 (Table 1, #13) and 1992 (Table 1, #14), and depredation from longline fisheries for Greenland halibut in 1970 (Table 1, #15) and 1971 (Table 1, #16).

### Marine mammals

#### Pilot whales (*Globicephala melas*, Traill, 1809)

Possible predation on long-finned pilot whales was reported once in the scientific literature (Table 1, #17).

#### Common minke whale (*Balaenoptera acutorostrata*, Lacepede, 1804)

Accounts of predation on minke whales, including confirmed consumption, were published in newspapers in 1915 (Table 1, #18) and 1997 (Table 1, #19).

**Fig 4:**
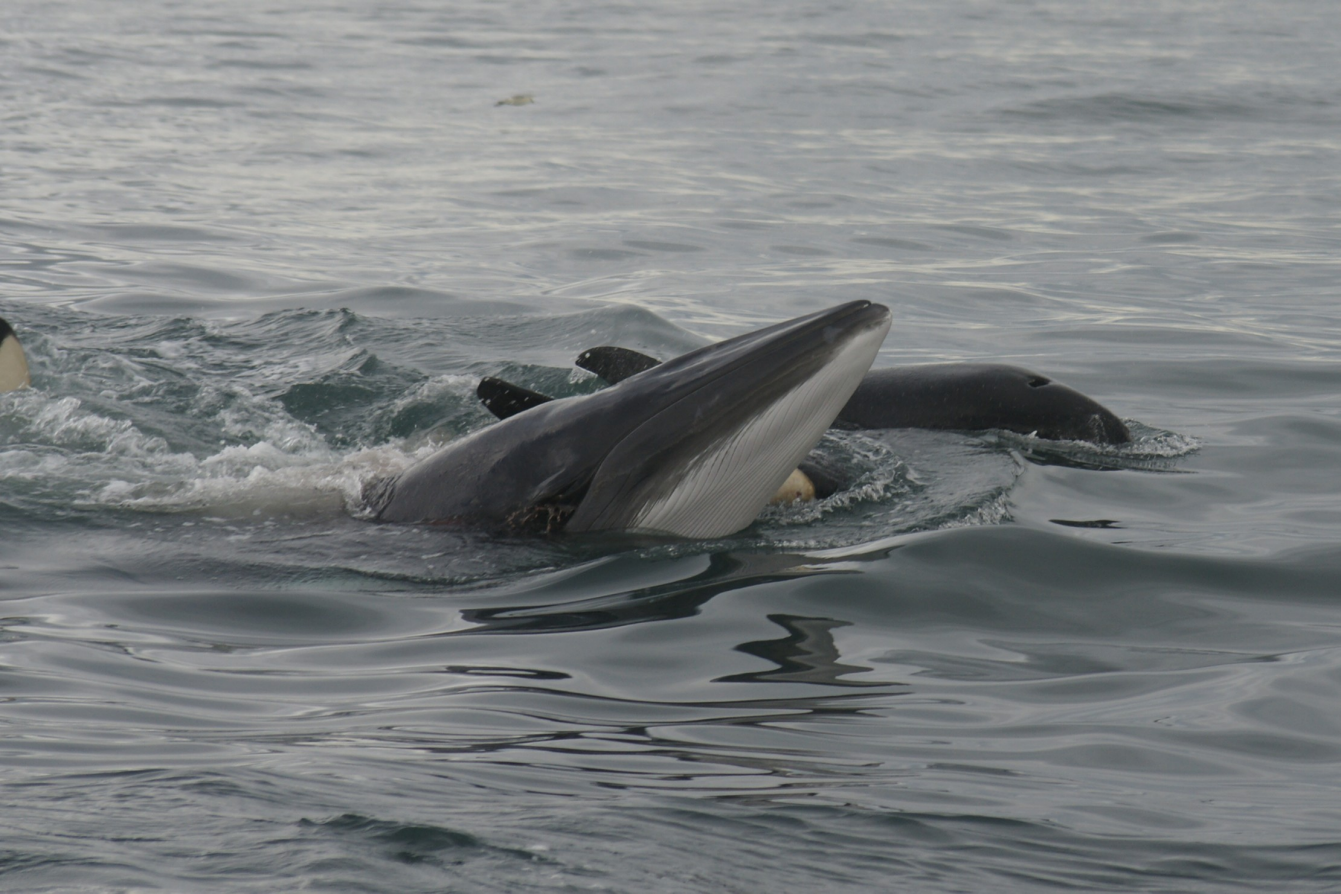
Killer whale predation on a minke whale. Observed in Skjálfandi bay (NE Iceland) on 22 July 2008 (photograph by M. Rasmussen).

On 22 July 2008 killer whales were observed in Skjálfandi bay off Húsavík (N Iceland; Table 1, #20). The sighting started with the observation of a common minke whale jumping out of the water. A whale-watching vessel headed in that direction to find a group of killer whales chasing the minke whale (Fig 4). The group consisted of approximately 20 individuals of all age/sex classes including one adult male. Two to four killer whales took turns surrounding the minke whale, but the adult male always stayed at a distance of more than 50 m and did not take part in the attack itself. The minke whale was observed jumping out of the water several times. Towards the end of the attack, the minke whale’s tongue was hanging out of its mouth and the killer whales dragged it underwater and kept it under the surface. The encounter lasted approximately 45 minutes, but consumption was not confirmed. The killer whales left the bay once the minke whale was dead. Photographs of some of the whales involved in this event were available but it was not possible to identify the individuals involved.

#### Harbour porpoise (*Phocoena phocoena*, Linnaeus, 1758)

Accounts of predation on harbour porpoises were published in newspapers in 1982 (Table 1, #21) and 1997 (Table 1, #19).

On 27 June 2012 a group of about five killer whales, including one adult male, was observed by whale-watching vessels attacking a harbour porpoise as well as a harbour seal (*Phoca vitulina*) in Skjálfandi Bay off Húsavík (Table 1, #22) within one hour. One killer whale was observed taking the harbour porpoise in its mouth, but consumption of both prey was not confirmed. The whales left the area soon after the hunt ended. Photographs from this event were available but not suitable to confirm matching for photo-identification.

**Fig 5:**
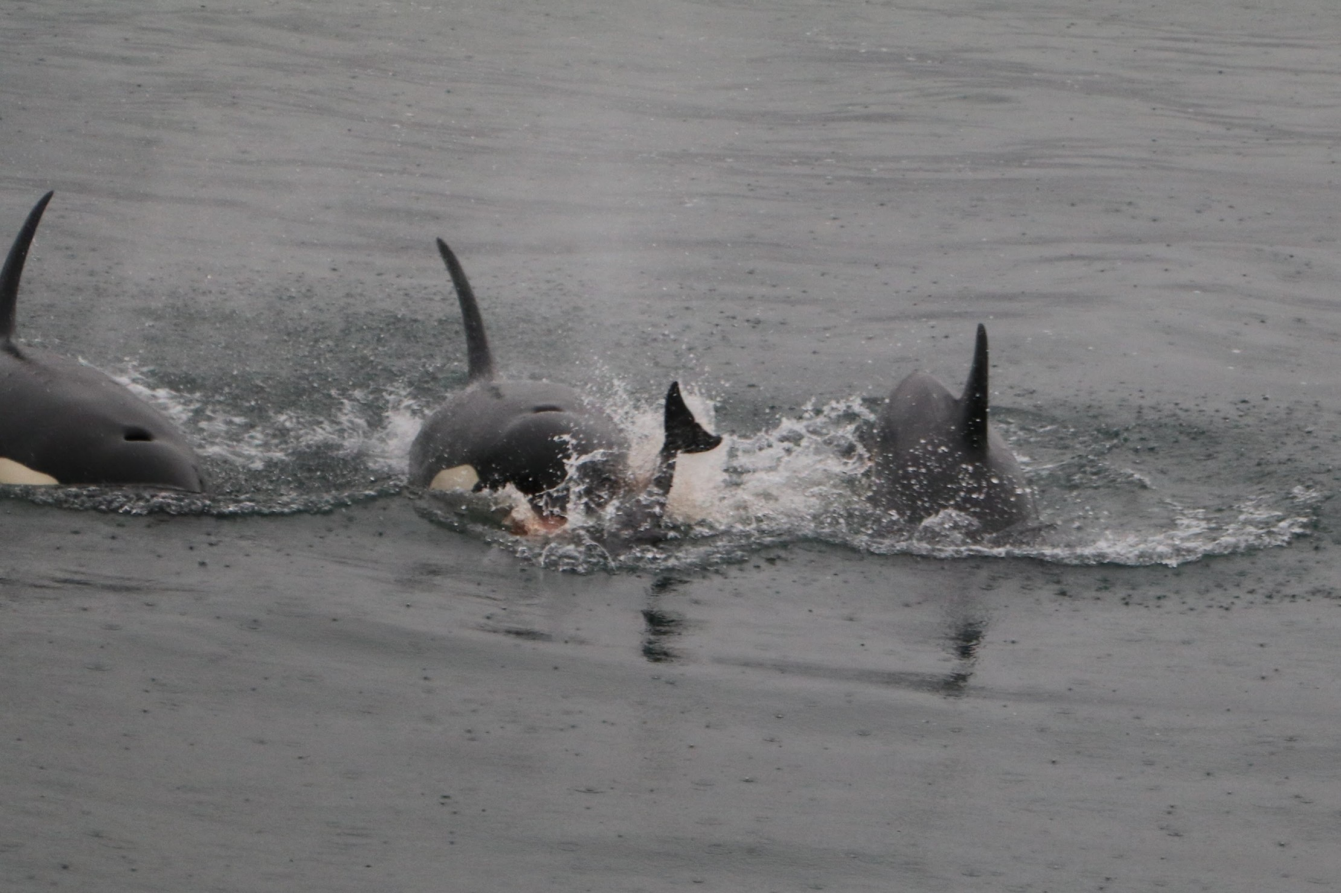
Killer whale predation on a harbour porpoise. Observed in Eyjafjörður (N Iceland) on 23 September 2017 (photograph by M. Ó. Elíasdóttir).

On 23 September 2017, at approximately 10:05 a group of killer whales was seen travelling in Eyjafjörður (Table 1, #23). The group was estimated as 12 individuals including two adult males and a young juvenile. Two humpback whales (*Megaptera novaeangliae*, Borowski, 1781) were also seen approximately 700 m away, nearer the coast, and a group of approximately 10–15 harbour porpoises was in the same general area, nearer the middle of the fjord. The killer whales travelled initially in a southern direction into the fjord, but then turned sharply and headed back to the area where the harbour porpoises were. Four to six killer whales, including an adult male, started speeding towards the harbour porpoises where they ended up making a kill (Fig 5). Following the kill, all whales seemed to join and consume the prey. At least one porpoise was killed, but the actual number taken is unknown. The killer whales then left the fjord, remaining close to the coastline. The encounter lasted approximately 50 minutes. Photographs of some of the whales involved in this event were available but it was not possible to identify the individuals involved.

#### White-beaked dolphin (*Lagenorhynchus albirostris*, Gray, 1846)

**Fig 6:**
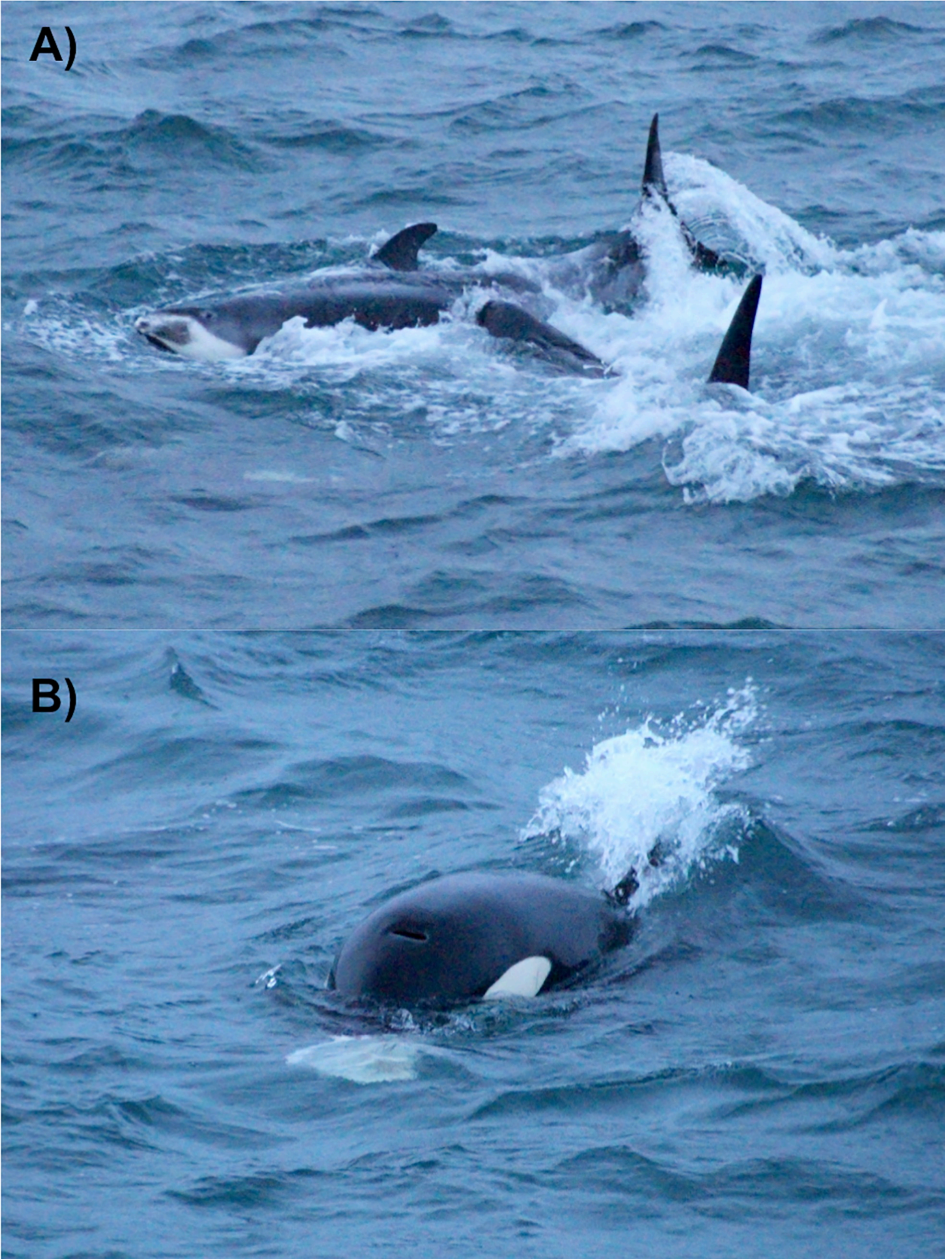
Killer whale predation on a white-beaked dolphin. Observed in Faxaflói bay (SW Iceland) on 17 December 2017 (photograph by J. Rempel): A) killer whale ramming the ventral side of the dolphin; B) killer whale holding a white piece of presumed dolphin blubber in its mouth.

On 17 December 2017, at approximately 15:30, a single white-beaked dolphin was observed from a whale-watching boat in Faxaflói Bay (Table 1, #24). This was followed by a sighting of a killer whale calf leaping out of the water nearby. Soon the rest of the killer whale group, making a total of five whales, appeared and surrounded the dolphin, with a lot of water splashing at the surface. The dolphin surfaced a few more times, then disappeared. Following the sighting of one of the adult whales with a large white ‘chunk’ in its mouth, the dolphin was presumed to have been successfully hunted (Fig 6). The whales then milled around the boat for some time. The encounter lasted approximately 25 minutes, and then the boat left the group. Photographs from this event were available but not of suitable quality to enable photo-identification.

#### Grey seal (*Halychoerus grypus*, Fabricius, 1791)

Predation on grey seals was published in newspapers in 1967 (Table 1, #25).

**Fig 7:**
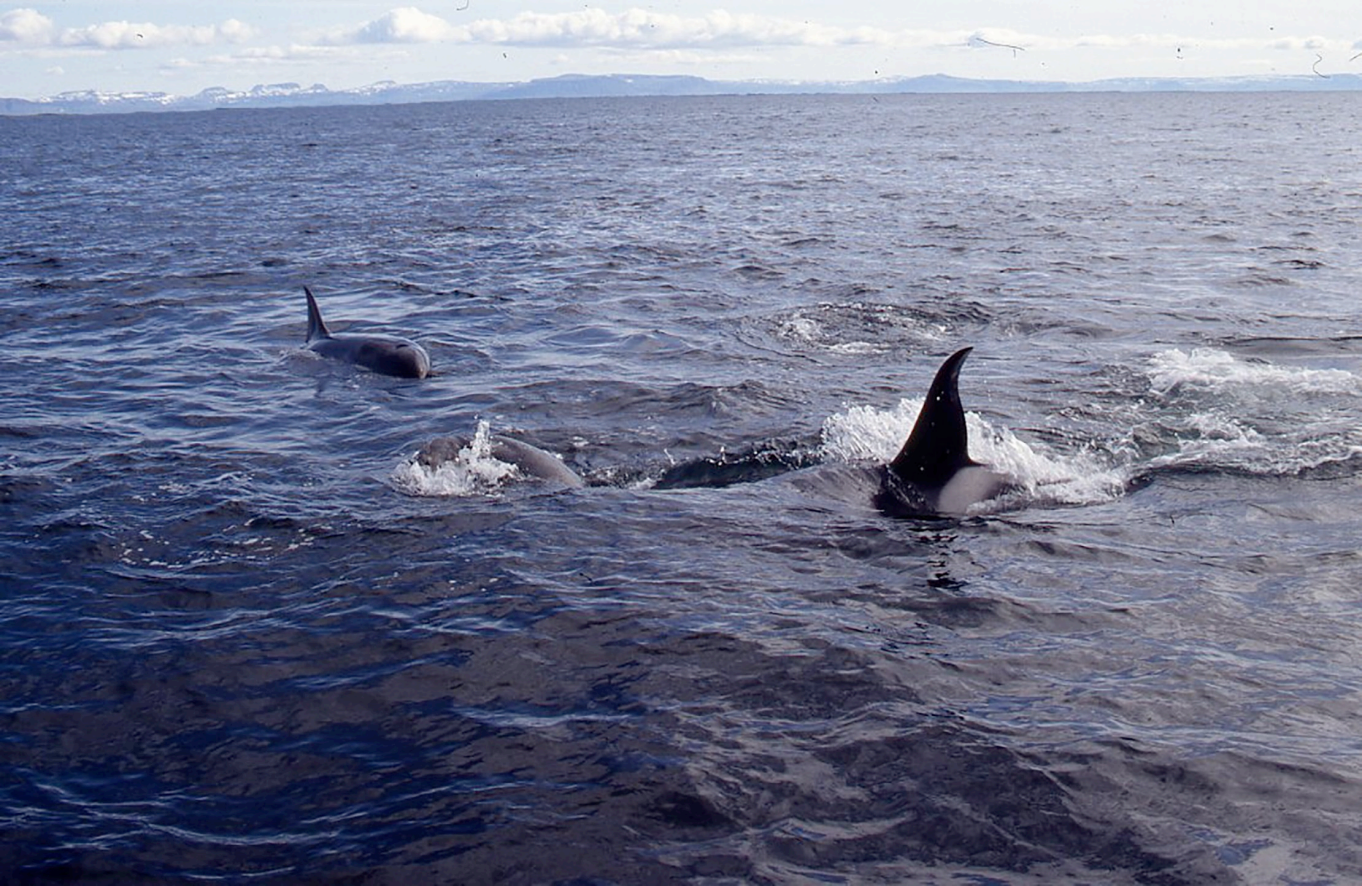
Killer whale predation on a grey seal. Observed in Breidafjörður (W Iceland) on 7 July 1993 (photograph by K. Gunnarsson).

On 7 July 1993, a group of seven killer whales including at least one adult male and one presumed calf, was sighted circling the shallows of Flóta-flögur in Breiðafjörður by observers on a small fishing boat (Fig 7; Table 1, #26). As a grey seal surfaced in the middle of the group, the killer whales increased their swimming speed and swam in a tighter circle. The seal then dove into an underwater kelp forest while the whales kept circling the shoals. After several surfacings by the seal, the whales took turns in attacking the seal, repeatedly ramming it and hitting it with their fluke. The whales repeatedly rammed and hit the seal and the seal could be seen clearly bleeding. The captain then took his shotgun and shot the seal when it surfaced. When the captain moved the boat towards the seal to try to hook it out of the water, one of the whales came alongside the boat from behind and grabbed the seal and swam off with it. The group joined in and a large red patch appeared on the sea surface. The event lasted for an estimated one hour and photographs were available but not of suitable quality for photo-identification.

#### Harbour seal (*Phoca vitulina*, Linnaeus, 1758)

Predation on a harbour seal, in the same event of predation on a harbour porpoise, was observed in Skjálfandi bay in 2012 (see above; Table 1, #27).

## Discussion

In this study we present records of 13 different species thought to be prey to killer whales occurring in Icelandic waters, including birds, cephalopods, fish and marine mammals. This list includes many but not all of the same prey reported in neighbouring regions of the North Atlantic (Table 2). All species except lumpfish, Atlantic halibut and Greenland halibut have been reported as killer whale prey in neighbouring regions. In contrast, a total of 13 species including birds (n = 5), jellyfish, fish (n = 3) and marine mammals (n = 4) have been reported in neighbouring regions but not in Iceland to date. The strandings network managed by the Marine and Freshwater Research Institute includes very few stomach contents from stranded whales and these have not been analysed yet. In addition, sub-surface consumption of fish, cephalopods or other prey is often difficult to determine. Thus, it is not unlikely that future work will increase the list of known prey in Icelandic waters.

**Table 2:** Summary of potential and actual prey items reported for killer whales in Iceland (this study) and a comparison with some neighbouring regions.

Prey type	Species	Iceland	East Greenland	Faroes	Norway	British Isles
Birds	Common eider (*Somateria mollissima*)	x		x^[^^40^^]^	x^[^^41^^]^	x^[^^42^^,^ ^43^^]^
	Northern fulmars (*Fulmarus glacialis*)				x^[^^41^^]^	
	Little auks (*Alle alle*)				x^[^^41^^]^	
	Kittiwake (*Rissa tridactyla*)			x^[^^40^^]^		
	Guillemot (*Uria aalge*)			x^[^^40^^]^		
	Puffin (*Fratercula arctica*)			x^[^^40^^]^		
Cephalopods	Squid (unknown spp.)	x			x^[^^44^^]^	
Cnidaria	Jellyfish (unknown spp.)				x^[^^41^^]^	
Fish	Herring (*Clupea harengus*)	x			x^[^^41^^,^ ^45^^]^	x^[^^46^^]^
	Lumpfish (*Cyclopterus lumpus*)	x				
	Salmon (*Salmo salar*)	x			x^[^^47^^]^	x^[^^48^^]^
	Atlantic halibut (*Hippoglossus hippoglossus*)	x				
	Greenland halibut (*Reinhardtius hippoglossoides*)	x				
	Cod (*Gadus morhua*)				x^[^^44^^]^	
	Saithe (*Pollachius virens*)				x^[^^41^^]^	
	Mackerel (*Scomber scombrus*)			x^[^^40^^]^	x^[^^41^^]^	x^[^^49^^]^
Marine mammals	Pilot whale (*Globicephala melas*)	x		x^[^^40^^]^		
	Common minke whale (*Balaenoptera acutorostrata*)	x	x^[^^50^^]^		x^[^^51^^]^	
	Harbour porpoise (*Phocoena phocoena*)	x		x^[^^40^^]^	x^[^^51^^]^	x^[^^52^^]^
	White-beaked dolphin (*Lagenorhynchus albirostris*)	x				x ^[^^53^^,^^54^^]^
	Bottlenose whales (*Hyperoodon ampullatus*)				x^[^^55^^]^	
	Sperm whale (*Physeter macrocephalus*)				x^[^^51^^]^	
	Grey seal (*Halychoerus grypus*)	x		x^[^^40^^]^	x^[^^56^^]^	x^[^^52^^]^
	Harbour seal (*Phoca vitulina*)	x			x^[^^56^^]^	x^[^^46^^,^ ^52^^,^ ^57^^]^
	Harp seal		x^[^^50^^]^			
	Hooded seal		x^[^^50^^]^			

The only bird species confirmed as prey of killer whales was the common eider. Eiders are very common in Iceland, with an estimated population size of ~850,000 and have a wide distribution [58], thus could be a reliable prey resource for killer whales. Common eider predation appears to occur regularly and is widespread, thus killer whales may be a significant predator of this species in Icelandic waters. However, it is unlikely killer whales rely heavily on this prey as a main source of food. Indeed, killer whales preying on birds may do so for “play” [59] or to help develop skills for hunting and handling prey [3].

Cephalopods are presumed to be an underappreciated component of the diet of transient killer whales and closely related ecotypes in the North Pacific Ocean [60]. We could only find one record of squid consumption based on stomach contents of a killer whale caught in the offshore waters east of Iceland [28]. Many records of cephalopod consumption by killer whales come from caught or stranded specimens where stomach contents have been collected [28, 60], indicating that visual observations of this behaviour are scarce, probably because they would take place at depth. Thus, it is possible that this behaviour occurs but simply has not been observed in Iceland.

Lumpfish, salmon, Atlantic halibut and Greenland halibut were the only fish species, other than the well-known herring prey, to be observed, although consumption was not confirmed in some cases. One of the interactions with lumpfish was by the same whales involved in an interaction with a common eider, suggesting these whales may target diverse prey. These killer whales are known to occur in Iceland in the winter apparently feeding upon herring but then travel to Scotland where they are regularly sighted in the spring and summer [20]. This indicates they do not specialize on the Icelandic summer-spawning herring stock year-round. Indeed, stable isotope measurements of IS015 and IS229 revealed inclusion of prey other than herring in their diet, suggesting these individuals have a varied diet relative to putative herring-specialists [19]. Lumpfish migrate from offshore feeding areas to coastal areas during the breeding season (approximately March to August) [61] and, thus, could be a prey seasonally available. In Iceland, lumpfish is also known to be an important part of the diet of sperm whales (*Physeter macrocephalus*, Linnaeus, 1758) [62].

One predation event involved the chasing and capture of a salmon in Vestmannaeyjar by individuals IS130, IS340, IS412 and a calf. These whales are regularly seen in the area and are believed to follow herring year-round because they are sighted in both herring overwintering and spawning grounds [21]. Vestmannaeyjar is a herring summer spawning ground [63], but also lies in the path of the Atlantic salmon’s yearly migration to the Rangá, Þjórsá and Ölfusá rivers in the south of Iceland. From spring to late summer, tens of thousands of salmon appear in these rivers, with estimates for the year of 2013 of around 12,000 salmon migrating to the Þjórsá river alone [64]. Although over several field seasons in Vestmannaeyjar this was the only confirmed event involving salmon, it cannot be excluded that this may be a prey targeted regularly. For example, for the majority of observed feeding events of ‘resident’ killer whales off the Northwest Pacific prey identification was achieved through sampling of tissues and scales rather than by direct observation of prey species [65]. At least 96% of these events were of predation on salmonids.

Atlantic halibut and Greenland halibut depredation from longlines were reported in newspapers, including accounts that fishermen had to give up the longline fisheries due to depredation by killer whales. Indeed, in 2002 a fisherman reported that the Greenland halibut fishery changed fishing gear to nets because using longlines failed due to depredation by killer whales and sperm whales in the east and west of Iceland, respectively [66]. The Atlantic halibut population in Icelandic waters decreased drastically during 1985–1992 and has been protected from direct fishing since 2012 [67]. On the other hand, Greenland halibut is one of the most abundant groundfish species inhabiting the waters west, north and east of Iceland [68]. Because the Greenland halibut fishery is nowadays largely undertaken by trawlers, with only some vessels using gillnet and longline [69], depredation may no longer be an issue at present.

As for marine mammals, a total of six prey species were reported in this study. Long-finned pilot whales have been previously recorded in both predatory and non-predatory interactions with killer whales [2]. In Iceland, we could find only one record of killer whales attacking pilot whales. In fact, interactions between pilot whales and killer whales have occurred often in recent years, but those are of pilot whales chasing killer whales off Vestmannaeyjar (Samarra, personal observation), as observed in Gibraltar [70] and Norway [71]. This suggests killer whale predation on pilot whales is not common in Iceland, however the interspecific dynamics of these species warrant further study.

In a study reviewing interactions of killer whales with marine mammal prey, minke whales were recorded as one of the most common cetacean prey species [2]. In addition to accounts of minke whale predation in local newspapers, in this study we report one incidence of an attack on a minke whale in Skjálfandi Bay where consumption was not confirmed. Surplus killing by killer whales, whereby prey is killed but not consumed, has been previously observed in various regions (see [2]) but the reasons for this behaviour are unknown [2]. The observation in Skjálfandi Bay described in this study resembles those of coordinated attacks by transient killer whales on minke whales in the Pacific, involving prolonged chases and ending in ramming and/or asphyxiation [72]. Further reports [22] and tooth rake marks observed on the skin of minke whales [73] suggest attacks on minke whales are likely to be common in Icelandic waters.

Harbour porpoises are commonly taken by killer whales in several locations [2]. The reports in this study confirm that predation on harbour porpoises also occurs in Iceland. The harbour porpoise is one of the most common small cetaceans in coastal Icelandic waters, however there is little information on population size and conservation status [74]. In this study we included four reports of harbour porpoise predation. However, the extent to which killer whales may predate on this species remains unknown. The fact that the killer whales involved in one of the predation events reported here were not matched to an existing photo-identification catalogue could suggest the occurrence of infrequently seen individuals that may belong to a separate, marine-mammal specialist ecotype. However, further predation records accompanied with photo-identification data will be necessary to assess this possibility.

Killer whales and white-beaked dolphins have been seen in non-predatory interactions in both Scotland and Iceland [2], and in Iceland the two species have been seen feeding together [18]. Killer whales have been observed hunting or attacking white-beaked dolphins in Scotland [53, 54] but detailed accounts of these events were not provided. In Iceland, skin lesions consistent with killer whale rake marks in white-beaked dolphins from Skjálfandi Bay and Faxaflói Bay suggested that killer whales are natural predators [73]. However, the rake mark prevalence was low [73, 75], indicating that interactions with killer whales may only occur occasionally. Our study reports the first observation of confirmed predation on white-beaked dolphins by killer whales in Iceland, but based on a single event supports a low prevalence of predation on this species.

Several seal species have been targeted by killer whales worldwide. Harbour seals appear to be one of the main seal prey [2], with some killer whales apparently specialising on this prey [76]. Only grey and harbour seals occur regularly throughout the year and breed in Iceland [77]. Both seal populations have wide distributions but have been declining in recent decades [77, 78]. The factors leading to population declines in Iceland are poorly understood, although hunting and bycatch, as well as prey availability are thought to be main contributors [78]. In this study, we included confirmed predation events on both seal species. Killer whales are thought to be the main natural predator of seals in Iceland [77] and further anectodal evidence suggests that predation on seals occurs more often than suggested by the few accounts presented here. However, assessing the predation pressure by killer whales on both seal species warrants further study.

This study shows that killer whales in Iceland take a wide range of prey. However, the extent of predation pressure on these prey species remains unknown and other potential prey items could remain undetected. Rake marks suggest interactions with other potential prey. For example, humpback whales sighted in Iceland exhibited a scarring rate of approximately 8% [79]. Predatory interactions are thought to occur in high-latitude waters [79], suggesting some of these events could happen in Iceland. A separate study reported a rake mark frequency in humpback whales of 13% in Icelandic waters and showed that generally predation of humpback whales by killer whales was largely confined to young animals [80], as seen in other locations [81]. However, to our knowledge, predatory interactions between humpback whales and killer whales in Iceland have not been documented to date. Tooth rake and bite marks have also frequently been observed on fin (*Balaenoptera physalus*, Linnaeus, 1758) and sei (*Balaenoptera borealis*, Lesson, 1828) whales in Icelandic waters (G. Víkingsson, personal observation). Although some killer whales that had stranded in Iceland grouped genetically with whales spatially associating and feeding upon mackerel [16], to date we have not found observations of mackerel predation. Thus, with an increased observer effort in the coastal waters of Iceland, the list of potential prey species may increase in the future.

The killer whale population in Iceland shows diversity in foraging strategies, with some whales apparently specialising upon herring while others include higher trophic level prey in their diet [19]. This is consistent with the existence of an ecological gradient where individuals and/or groups vary in the proportion of different prey items consumed, as suggested for Type 1 killer whales [15]. Given our observations of interactions with marine mammals, we cannot exclude the possibility that marine mammal specialist killer whales [15] occur in Icelandic waters. Some of the whales observed off Húsavík attacking a minke whale had narrow and fainter coloured saddle patches, which were similar to those described in other regions (Hawaii [82]; Caribbean [83]; Gulf of Mexico [84]) but unlike most saddle patches in an existing photo-identification catalogue [24]. However, this feature was not shared among all individuals involved in the event suggesting they were not of a single morphotype. Killer whales with diets presumed to include herring and other high trophic level prey were observed interacting with a lumpfish and a common eider, supporting a diverse diet in these individuals. On the other hand, killer whales believed to feed predominantly on herring were observed interacting with salmon. Prey specialisation does not imply exclusivity, as seen by ‘resident’ killer whales in the NE Pacific that specialise on salmon but occasionally take other fish [3]. However, it is also known that killer whales often kill prey they do not intend to eat [59] and we cannot rule out the possibility that it occurred in some of the events we describe. Clearly, continued observations will be essential to assess the frequency by which different prey items are consumed and evaluate their contribution to the diet of individuals/groups observed in Iceland.
